# Technical Considerations and Protocol Optimization for Neonatal Salivary Biomarker Discovery and Analysis

**DOI:** 10.3389/fped.2020.618553

**Published:** 2021-01-26

**Authors:** Elizabeth Yen, Tomoko Kaneko-Tarui, Jill L. Maron

**Affiliations:** ^1^Mother Infant Research Institute at Tufts Medical Center, Boston, MA, United States; ^2^Division of Newborn Medicine, Tufts Children's Hospital, Boston, MA, United States

**Keywords:** saliva, neonate, transcriptome, proteome, biomarker

## Abstract

Non-invasive techniques to monitor and diagnose neonates, particularly those born prematurely, are a long-sought out goal of Newborn Medicine. In recent years, technical advances, combined with increased assay sensitivity, have permitted the high-throughput analysis of multiple biomarkers simultaneously from a single sample source. Multiplexed transcriptomic and proteomic platforms, along with more comprehensive assays such as RNASeq, allow for interrogation of ongoing physiology and pathology in unprecedented ways. In the fragile neonatal population, saliva is an ideal biofluid to assess clinical status serially and offers many advantages over more invasively obtained blood samples. Importantly, saliva samples are amenable to analysis on emerging proteomic and transcriptomic platforms, even at quantitatively limited volumes. However, biomarker targets are often degraded in human saliva, and as a mixed source biofluid containing both human and microbial targets, saliva presents unique challenges for the investigator. Here, we provide insight into technical considerations and protocol optimizations developed in our laboratory to quantify and discover neonatal salivary biomarkers with improved reproducibility and reliability. We will detail insights learned from years of experimentation on neonatal saliva within our laboratory ranging from salivary collection techniques to processing to downstream analyses, highlighting the need for consistency in approach and a global understanding of both the potential benefits and limitations of neonatal salivary biomarker analyses. Importantly, we will highlight the need for robust and stringent research in this population to provide the field with standardized approaches and workflows to impact neonatal care successfully.

## Introduction

For decades, neonatologists have recognized that saliva provides a window into their vulnerable patients' physiology and pathophysiology (1, 2). While initial studies focused on quantitative cortisol levels with corresponding stress response (3, 4), more recent studies have demonstrated that salivary analyses can provide insight into development through both global and targeted gene and/or protein expression analyses (5–7). Many of the technical limitations that previously restricted neonatal salivary research have been overcome, including the need for robust saliva volumes for analyses. However, new challenges for the field must now be addressed. Proper acquisition of samples, processing and stabilization, and knowledge regarding downstream analytic platforms must be standardized for successful integration. Understanding the impact of salivary microbial content on platforms such as RNA sequencing (RNASeq), and how to circumvent potential contamination is needed. As the field continues to evolve toward clinical translation, it is imperative that researchers and clinicians recognize the potential benefits and limitations of saliva as a non-invasive biofluid for biomarker discovery and neonatal assessment. Only then will we be able to non-invasively and accurately assess our patients and personalize treatment strategies.

### Neonatal Salivary Biomarkers

Biomarkers may be well-known and clinically accepted by clinicians (e.g., c-reactive protein [CRP]) or newly discovered and found to be recently linked to a clinical outcome (e.g., neuropeptide Y2 receptor [NPY2R]) (6). Each type of biomarker presents challenges for the investigator. For example, established biomarkers, such as cytokines, are often already well-accepted by the medical community as clinically relevant with established reference ranges in plasma or serum. Attempting to use saliva for known biomarker quantification involves extensive comparative analyses between blood and saliva to demonstrate that saliva is as valid a source of biomarker information as blood (8, 9). Conversely, novel biomarkers typically lack blood correlate data and thus, do not require comprehensive comparative biofluid analyses. One need not assume that biomarker detection in the blood is superior to other biofluids. In fact, certain biomarkers such as cytomegalovirus (CMV) have been shown to be more reliable in saliva (9, 10). Rather, the challenge with novel biomarkers lies in convincing the medical community that the salivary biomarker itself will aid in clinical decision making. Here, the investigator must perform hundreds, if not thousands, of salivary analyses to account for multiple confounding variables to demonstrate that the novel salivary biomarker will inform care.

**Figure 1:**
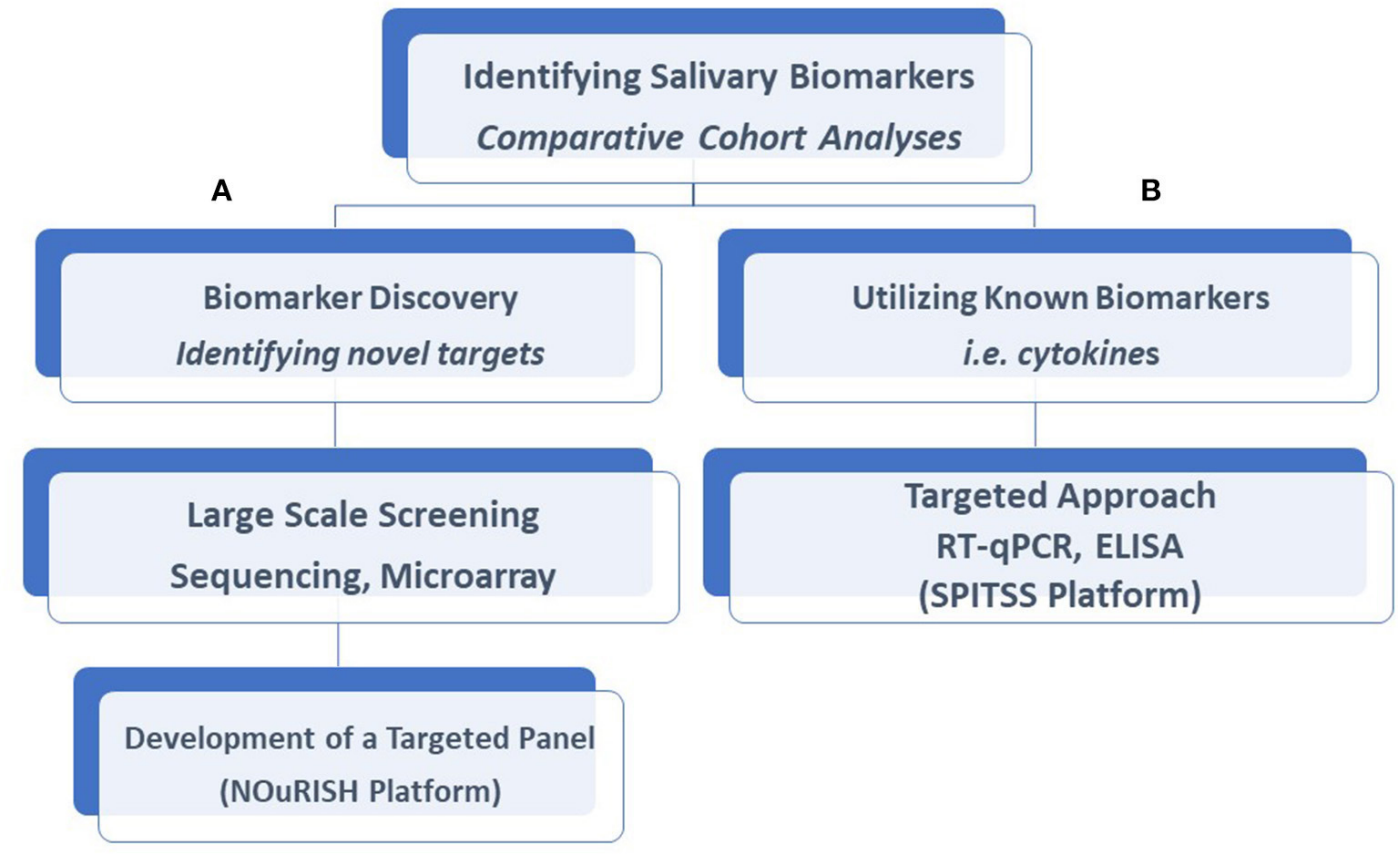
Salivary biomarkers discovery and analysis. Our laboratory had taken a two-tiered approach to the identification of salivary biomarkers and the development of diagnostic assays. Comparative analyses of salivary profiles between infants affected by a variety of disorders with unaffected control infants allows us to identify novel biomarkers with the use of large-scale screening platforms described in this article, such as the NOuRISH platform **(A)** or assess the accuracy of known biomarkers, such as the SPITSS Trial **(B)** in making a diagnosis. Both proteins and gene targets (mRNA) are amenable to these approaches. RT-qPCR, Reverse Transcription-qualitative Polymerase Chain Reaction; ELISA, Enzyme-Linked Immunosorbent Assay; SPITSS, Salivary Profiling in Infants Treated for Suspected Sepsis; NOuRISH, Neonatal Oral Feeding-readiness In Salivary High-throughput.

Despite the daunting task of conducting such large trials, progress is forthcoming. Large, multi-center trials funded by the NIH are currently underway exploring the clinical utility of both known and unknown neonatal salivary biomarkers (11). The Salivary Profiling in Infants Treated for Suspected Sepsis (SPITSS) Trial is serially quantifying and validating six salivary biomarkers (CRP, procalcitonin, interleukins [IL]-1ß, 6, 8, and TNF-α) in 4,000 infants across the United States (US) treated for a suspected infection. Salivary protein cytokine profiles will be correlated to clinical outcomes to determine if a salivary diagnostic panel can more rapidly and accurately discriminate between an infected and non-infected neonate compared to traditional biomarkers (e.g., blood culture) to reduce unnecessary antibiotic exposure. In a separate trial, a salivary diagnostic panel coined NOuRISH (Neonatal Oral Feeding-readiness In Salivary High-throughput diagnostics) composed of five novel mRNA biomarkers (*NPY2R, AMPK, PLXNA1, NPHP4*, and *WNT3*) related to oral feeding maturation is being tested in extremely premature neonates across several US hospitals (11, 12) (Figure 1). As data emerge from these trials and others, it is crucial to recognize that standardized approaches to saliva collection, processing, and analyses are mandated for proper interpretation. Failure to institute such procedures will only serve to undermine the field and its progress.

### Salivary Collection

The majority of commercially available saliva collection devices depend upon active or passive drool acquisition following the rinsing of the oropharynx prior to sample collection. Alternative approaches involve saliva collection via absorption from sponge or wick devices. Unlike adults with ease of collection and generous salivary volume, salivary acquisition in newborns could be challenging due to the minimal amount of saliva they generate. However, with the recognition of the vast applications for salivary research in the neonate, collection techniques now incorporate miniaturized sponge applicators and pacifier collection devices with a self-absorbing wick for use in the newborn, infant, and toddler.

For over a decade, our laboratory has utilized a gentle bedside suction technique to collect saliva samples. The methodology for this approach has been published previously (13) and has been applied to neonates as young as 24 weeks' gestation age and as small as 400 grams Briefly, the plunger of a 1 mL syringe is removed, and the syringe itself is attached to low bedside suction (not exceeding 10 mm Hg negative suction pressure) following the removal of its endcaps to allow for direct attachment to suction tubing (i.e., the end of the syringe is cut into a circular shape to permit attachment to tubing). The infant's oropharynx is gently suctioned for 20 to 30 s, in areas where saliva is known to pool, including under the tongue and in between the cheek and gums. Following collection, the plunger is placed back into the syringe and used to aspirate and release the saliva into a stabilizing solution directly. On average, suctioning of the mouth yields between 10 and 50 μL of the whole saliva (14). Sponges and wick applicators will yield slightly more volume, directly correlated to collection time. It is strongly recommended that duplicate or triplicate of saliva samples be collected at any time point from each neonate. Additional samples can be used in a pooled fashion to increase sample volume needed for analysis or used as an alternative sample in the case of assay failure. For a diagram of the suctioning methodology using the syringe, please refer to an article by Dietz et al. (13).

As saliva production is directly related to the infant's hydration status, full-term infants at the nadir of their weight loss are challenging. Thus, sponge placement or pacifier use may need to be modified accordingly by alternating the placement sites of the sponge in the mouth or by placing the pacifier inside the mouth for a longer time. Conversely, the act of suctioning itself often stimulates saliva production in these newborns. Slightly longer suction time at the bedside typically produces adequate volume necessary for analysis.

### Salivary Processing and Stabilization

It is essential that saliva be stabilized immediately after collection to prohibit biomarker destruction degrative enzymes present in the oropharynx. The volume of stabilizing agent containing protease and RNase inhibitors should be adjusted according to the expected sample volume collected in the neonate. Stabilizing volume can be reduced 5- to 10-fold from the manufacturer's recommendations for use in the neonatal population. Jiang et al. have described a universal stabilizing cocktail for use with salivary proteins, RNA, and DNA (15) that has shown success in the neonatal population (8). Sponge applicants may be frozen after collection if immediate centrifugation for sample retrieval is not possible.

### Normalization

Given the dependence of saliva production on overall hydration status, we do not recommend normalization based upon volume. Rather, normalization must be done with reference genes and proteins that account for the rapid, ongoing development in the premature and term neonatal population. Reference genes and proteins must demonstrate stable expression across development, age, and sex. Previously, our laboratory analyzed 360 saliva samples across a wide range of gestational and post-menstrual ages to determine that *GAPDH, YWHAZ*, and *HPRT1* (16) maintain their stability in this patient population. We mandate the expression of all three reference genes in a given sample to ensure that the relative quantification of the target gene(s) of interest is reliable. Failure for a sample to amplify all reference targets results in elimination from the analysis.

### Quality Assessment

Biomarkers in neonatal saliva are inherently of poor quality. Quality assessment platforms for sample integrity determination prior to downstream analyses allow investigators to select the best samples for use. The NanoDrop microvolume spectrometer and fluorometer can quantify starting amounts of DNA, RNA, and proteins targets to picomole concentrations. Similarly, the Agilent Bioanalyzer 2100 can quantify and qualify RNA integrity down to the picomole level and has been utilized in transcriptomic analyses. Total human RNA assessment is qualified based on detecting the unique 18s and 28s ribosomal RNA concentrations. The ratio between peaks is used to determine an RNA integrity number (RIN). A RIN of ≥ 7 indicates excellent quality, cellular RNA (17, 18). However, for a salivary biomarker assessment, the targeted RNA species are cell-free. Cellular content would largely represent gene expression occurring in buccal or tongue epithelial, not systemic gene expression released in the cell-free form. Thus, RINs of ≥ 7, while considered excellent quality RNA, are not of sufficient quality for cell-free salivary analyses. Rather, RINs of 2 to 4 are preferred to ensure that targets analyzed reflect systemic gene expression. Combining these qualitative assays with normalization via stable reference genes or proteins ensures that biomarkers identified and quantified are of the highest quality to assess systemic neonatal development, physiology, and pathology.

### Microbiome

The oropharynx is a rich source of symbiotic and potentially pathogenic microorganisms. Bacteria, fungi, and viruses are plentiful. Unique to the newborn is the rapid colonization of the oropharynx. In the premature newborn, this colonization is largely aberrant, modified by prematurity, delayed enteral feedings, the presence of non-sterile tubing, and prolonged hospitalizations. As such, the microbiome itself holds the potential to serve as a rich source of biomarkers that may predict impending morbidity in the neonate. Thus, salivary analyzes exploring both the identification of microbial profiles (16sRNA analyses), as well as their gene expression and function (RNASeq), may offer an opportunity to identify novel microbial biomarkers to enrich clinical assessment. Of note, unique ecosystems exist in the oropharynx, whereby microbial profiles differ throughout the mouth (19). Once again, a consistent approach to saliva collection is essential to achieve reproducible and reliable results.

## Diagnostic Platforms

### Proteomic Analyses

With proper stabilization, saliva's vast proteomic profiles can be assessed on either targeted or broad-based discovery platforms for biomarker discovery. The sensitivity of targeted protein assays is becoming more exquisite, capable of quantifying biomarkers at a molecular level (20). Further, the assays' rapid turnaround time, ranging from mere minutes to hours, may provide rapid integration of diagnostic platforms into care. Following the necessary federal regulatory assessments, diagnostic assays may be housed within a central laboratory under Clinical Laboratory Improvement Amendments (CLIA) oversight to provide real-time results. It remains imperative that investigators adhere to proper collection, stabilization, and normalization techniques to ensure reproducibility of results, confidence in clinical applicability, and integration into care.

### Reverse Transcription-Qualitative Polymerase Chain Reaction (RT-qPCR)

RT-qPCR offers multiplex salivary biomarker quantification. Assay sensitivity is limited to the number of amplification cycles to limit primer-primer interactions and the generation of contaminating amplicons. It has been our laboratory's experience that the inherent quantification limitations of the platform, combined with minute starting quantities of mRNA targets, biomarkers often fail to cross the threshold of amplification. This results in genes being interpreted in a binary fashion, i.e., positive or negative genes expression. This approach has both benefits and pitfalls. As one considers the commercialization of diagnostic platforms for biomarker detection, the simple detection of a gene's presence or absence related to a clinical phenotype allows for ease of interpretation. Not burdened with the complexities of quantitative levels, a caregiver can quickly assess and interpret the assay results, similar to ßhCG detection in a pregnancy test.

If an investigator prefers quantitative biomarker levels, or conversely, the biomarker itself is more accurately interpreted by relative quantification analyses between samples, a targeted pre-amplification may be performed (21). This targeted approach avoids universal amplification that will be inherently biased against degraded mRNA transcripts found in saliva samples. Custom pre-amplification reagents are commercially available and may be manufactured for particular targets of interest. Our laboratory has successfully employed this technique to adapt the interpretation of biomarker levels from binary to relative quantification (22).

### Gene Expression Microarrays

Our initial research on neonatal salivary diagnostics and biomarker discovery employed comparative analyses of salivary profiles over time using Affymetrix gene expression microarrays. We demonstrated that an enormous amount of real-time developmental information representing systemic biology, including neurodevelopment, could be monitored through this technique (5). One limitation of gene expression microarrays is that predominant gene transcripts can flood the system, limiting the detection of rarer transcripts in a given sample (23). To determine if cellular, whole saliva compared to cell-free salivary supernatant performed differently on gene expression microarrays, we performed comparative analyses of saliva samples obtained from the same infants simultaneously. While salivary supernatant analyses did identify expression of genes not seen in the whole saliva, overall, there was a 92.5% concordance in expression profiles (13). We speculate that the abundance of keratin transcripts ubiquitously expressed in the oral epithelial impacted the hybridization of rarer transcripts in the whole saliva, resulting in the discrepant results. Nevertheless, these data have demonstrated that neonatal saliva samples, are amenable to comprehensive high-throughput platforms and may be utilized in this patient population for biomarker discovery.

## RNA Sequencing

RNASeq has become the preferred method to measure gene expression due to its effectiveness and high throughput, largely replacing the lower-cost methods, e.g., northern blots and quantitative PCR. However, this technique requires *a priori* knowledge of the sequences of interest and has a limited ability to detect low-quality gene transcripts (24). High-throughput next-generation sequencing (NGS)-based technique can detect novel gene expression, non-coding RNAs, and alternative splicing information. RNASeq has been used to identify novel biomarkers in saliva (25–27), but technical issues and optimization for the neonatal population remain challenges to be solved. Unlike RT-qPCR and microarray platforms designed to be human-specific, RNASeq indiscriminately identifies gene transcripts, independent of the source. Thus, when analyzing biofluids containing a mixed source of gene input of the host and of the resident oral bacteria protocol adjustments need to be made.

### Selection of RNA Types and Library Preparation

Library preparation kits are chosen both to optimize the selection of RNA species to be sequenced (e.g., coding vs. non-coding) and to enhance gene detection. For instance, ribosomal RNA (rRNA) accounts for more than 90% of the total cellular RNA and can interfere with the quantification of gene transcripts. To reduce rRNA and increase the detection of less abundant RNAs, one could either reduce rRNA content with commercially available kits, such as RiboMinus (Life Technologies) or RiboZero (Epicenter), or enrich for coding RNA transcripts with the selection of polyadenylated (poly-A) RNAs (23). Transcript fragments are then converted to cDNA and amplified.

### RNASeq Data Analysis

There are three main steps involved in RNASeq analysis: (1) alignment of the RNASeq reads to a reference transcriptome or genome; (2) assembly of the aligned reads into transcription units (reconstruction of a transcriptome); (3) performance of differential expression of transcripts across conditions or time points (28). Investigators may choose the sequencing platform, the human genome (hg) to align data, and the bioinformatics software programs to analyze and interpret results. In general, it is expected that between 70 and 90% of regular RNASeq reads will map onto the human genome, with a slightly lower percentage for mapping against transcriptome (29).

**Figure 2:**
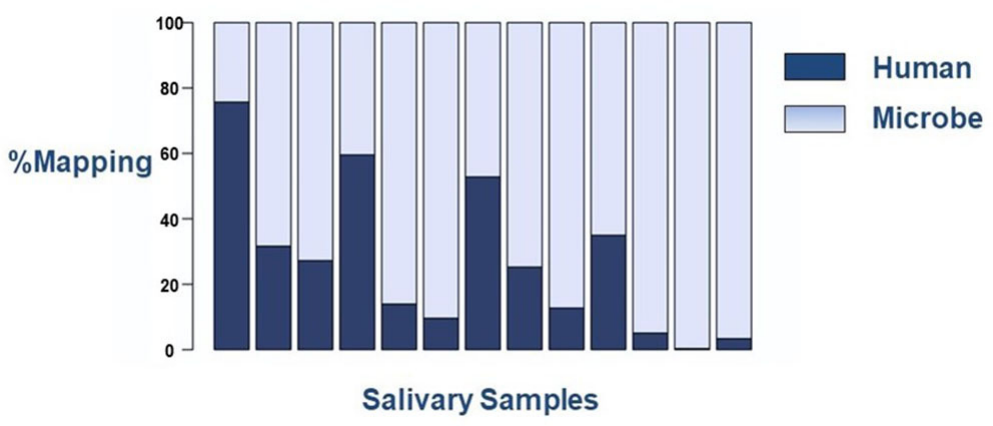
Sequencing mapping alignment rates to human or microbial genomes on the RNASeq platform. Each column represents an individual saliva sample. There is wide variation in alignment to the human genome between samples.

We have encountered two technical challenges when using RNASeq for novel salivary biomarker discovery in the neonate. The first is the abundance of prokaryotic microbes in saliva samples, which deleteriously interfere with sequencing read alignments (Figure 2). While this finding serves as evidence of the abundance of the oral microbiome in human saliva, even as early as few hours to days after birth, it presents a technical challenge to the field. Varying human alignment rates, certainly in excess of 30%, can result in unreliable results that cannot be used for meaningful comparisons between samples. One way to circumvent this limitation is to enrich the poly-A tail of mRNA targets during library preparation. Polyadenylation is unique to eukaryotic cells and does not occur in prokaryotic microbes. Thus, one can select only human transcripts for analyses on RNASeq, even if the starting source is of mixed origin.

**Figure 3:**
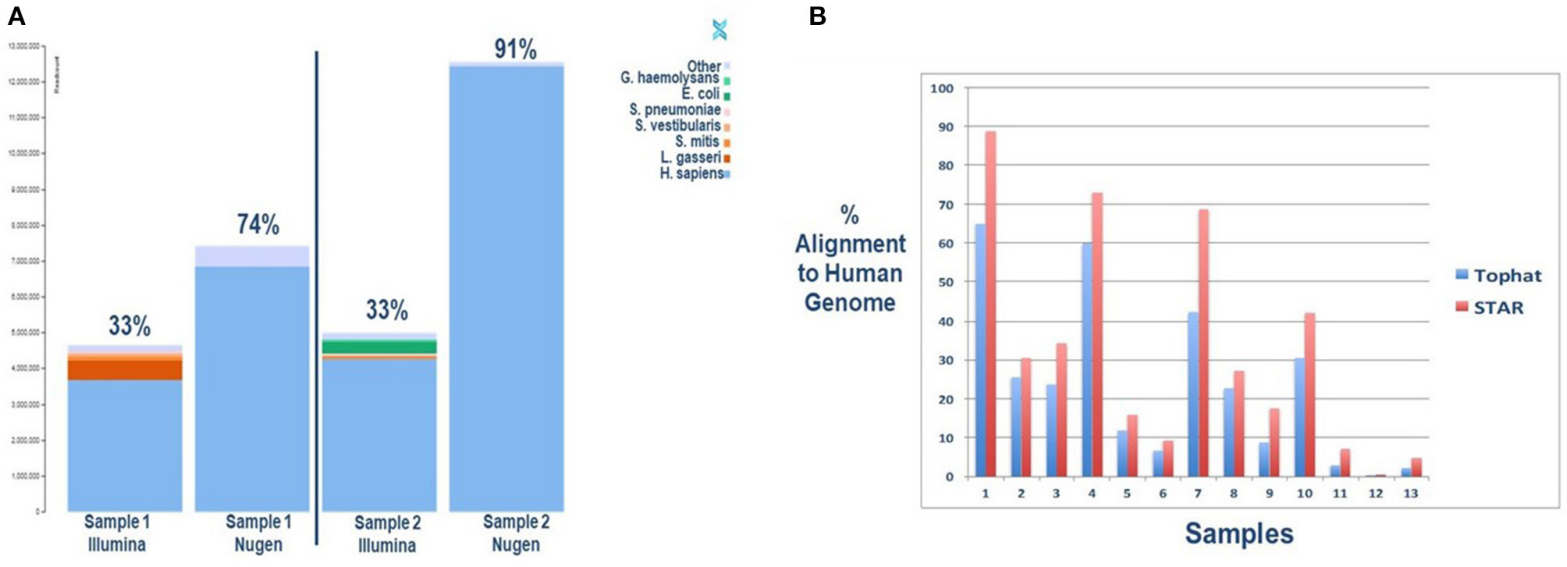
**(A)** Improved sequence mapping to the human genome with selection of polyadenylation with the Nugen library preparation kit compared to Illumina. Two salivary samples were collected simultaneously from two infants for comparative analyses. The key in the upper right corner provides species identification. **(B)** Improved sequence mapping to the human genome with STAR software, a “splicing-aware” aligner, compared to TopHat. A single sample was sequenced from each subject and aligned twice to the human genome with each respective software program.

To assess the impact of a targeted library preparation on reading alignments, we compared library preparation kits that enrich the poly-A tail (Nugen) to non-selective library preparation (Illumina). All samples underwent ribosomal RNA reduction with Ribo-Zero-Seq. The results are depicted in Figure 3A. The overall human alignment was significantly improved using the poly-A selective Nugen preparation kit, with a significant overlap in gene detection between kits (Supplementary Figure 1). Unsurprisingly, the Illumina kit was able to identify more RNA species, given its universal approach. Of note, by selecting out polyadenylation, the ability to detect for non-coding RNAs is lost. Thus, the investigator is limited to gene expression analyses in lieu of a more comprehensive analysis of regulatory RNAs.

The second technical issue confronted with RNASeq analysis of neonatal saliva is the potential for increased alternative splicing of transcripts, limiting read alignment to the human genome. Rapid development and tissue differentiation occurring in premature and term neonates result in alternative splicing of gene transcripts. Computational software has been developed to address increased gene splicing in a given sample. STAR is one of the available “splicing-aware” aligners specifically design to address this challenge (30). To determine if selective software improves alignment rates, we compared a commonly used aligner, TopHat, to STAR software. The results of this analysis are depicted in Figure 3B. Here, too, we can see an improved human genome alignment rate when one considers a developing newborn's unique biology. Methods for each approach are illustrated in Supplementary Figure 2 and serve to educate the investigator on novel approaches to address and overcome the challenges associated with biomarker discovery in neonatal saliva.

### Study Design Considerations

While consideration of technical limitations is essential to move the field of neonatal salivary biomarker discovery and diagnostics forward, so, too, is proper study design. Controlling for unique variables such as gestation and post-conceptional age, along with sex, race, and ethnicity, is required to establish accurate normative reference ranges for novel biomarkers and to appropriately interpret aberrant values.

## Conclusion

Over the past decade, our laboratory has worked diligently and methodically to address and overcome technical challenges associated with neonatal salivary biomarker analyses. We have demonstrated the feasibility, applicability, reliability, and reproducibility of neonatal salivary diagnostics and strive to show the potential impact non-invasive salivary analysis may have on the field. Utilizing state-of-the-art technologies, we have optimized methods on neonatal saliva collection, processing, sex- and gestational-age-appropriate reference gene selection. Our laboratory's exciting work serves as the foundation for ongoing research and robust exploration to benefit this vulnerable population. Further research, funding, and collaborations are needed to provide this population with the most advanced, innovative, non-invasive, and clinically relevant research.

## Data Availability Statement

The datasets presented in this article are not readily available because Data presented are only related to alignment rates. No specific gene or identifiable data are provided. Requests to access the datasets should be directed to jmaron@tuftsmedicalcenter.org.

## Ethics Statement

The studies involving human participants were reviewed and approved by Tufts Medical Center Institutional Review Board. Written informed consent to participate in this study was provided by the participants' legal guardian/next of kin.

## Author Contributions

EY and JM: paper concept and writing of manuscript. TK-T: technical assistance and manuscript editing. All authors contributed to the article and approved the submitted version.

## Conflict of Interest

The authors declare that the research was conducted in the absence of any commercial or financial relationships that could be construed as a potential conflict of interest.
