# Supplementary material for: Technical Considerations and Protocol Optimization for Neonatal Salivary Biomarker Discovery and Analysis
Source: Front Pediatr. 2021 Jan 26;8:618553. doi: 10.3389/fped.2020.618553 (PMC7870796; doi:10.3389/fped.2020.618553)
Supplement: Supplementary file 1 [file Data_Sheet_1.docx]

**Supplementary Files:**

**Supplemental Figure 1: Gene transcripts identified and their overlap using the Illumina and Nugen library preparation kits. Two saliva samples were analyzed. While there is a substantial overlap in gene detection, Illumina identified more gene targets.**


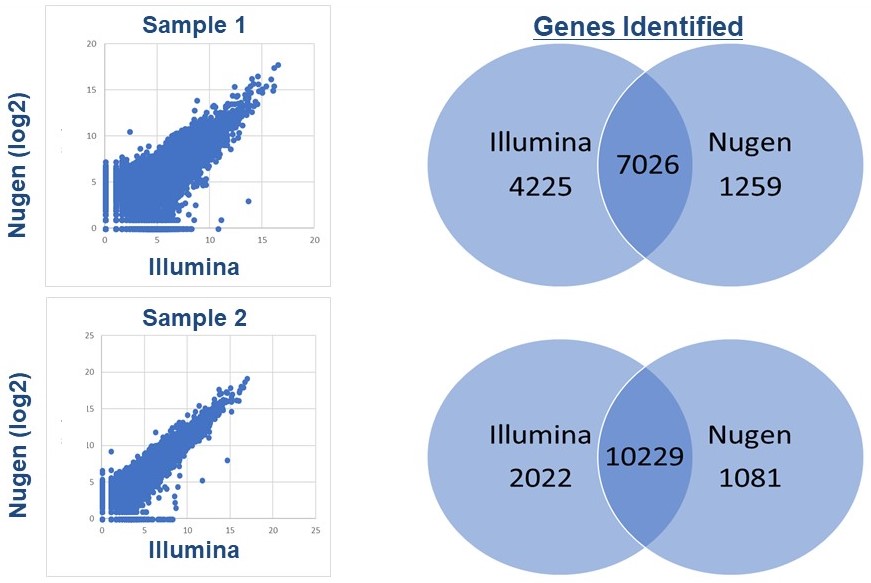


**Supplemental Figure 2: A schematic of the methodology to assess varying library preparation kits (a) and varying alignment software (b) to improve performance of saliva samples on the RNASeq platform.**
